# Features of a Self-Mixing Laser Diode Operating Near Relaxation Oscillation

**DOI:** 10.3390/s16091546

**Published:** 2016-09-21

**Authors:** Bin Liu, Yanguang Yu, Jiangtao Xi, Yuanlong Fan, Qinghua Guo, Jun Tong, Roger A. Lewis

**Affiliations:** 1 School of Electrical, Computer and Telecommunications Engineering, University of Wollongong, Northfields Ave, Wollongong, NSW 2522, Australia; bl987@uowmail.edu.au (B.L.); jiangtao@uow.edu.au (J.X.); qguo@uow.edu.au (Q.G.); jtong@uow.edu.au (J.T.); 2 School of Physics, University of Wollongong, Wollongong, NSW 2522, Australia; yf555@uowmail.edu.au (Y.F.); roger@uow.edu.au (R.A.L.)

**Keywords:** self-mixing effect, laser diode, relaxation oscillation, stability boundary

## Abstract

When a fraction of the light reflected by an external cavity re-enters the laser cavity, both the amplitude and the frequency of the lasing field can be modulated. This phenomenon is called the self-mixing effect (SME). A self-mixing laser diode (SM-LD) is a sensor using the SME. Usually, such LDs operate below the stability boundary where no relaxation oscillation happens. The boundary is determined by the operation condition including the injection current, optical feedback strength and external cavity length. This paper discovers the features of an SM-LD where the LD operates beyond the stability boundary, that is, near the relaxation oscillation (RO) status. We call the signals from such a SM-LD as RO-SM signals to differentiate them from the conventional SM signals reported in the literature. Firstly, simulations are made based on the well-known Lang and Kobayashi (L-K) equations. Then the experiments are conducted on different LDs to verify the simulation results. It shows that a RO-SM signal exhibits high frequency oscillation with its amplitude modulated by a slow time varying envelop which corresponds to the movement of the external target. The envelope has same fringe structure (half-wavelength displacement resolution) with the conventional SM signals. However, the amplitudes of the RO-SM signals are much higher compared to conventional SM signals. The results presented reveal that an SM-LD operating near the RO has potential for achieving sensing with improved sensitivity.

## 1. Introduction

In 1968, it was reported that when a fraction of the light back-reflected or backscattered by a remote target is allowed to re-enter the laser cavity, both the amplitude and the frequency of the lasing field can be modulated [1]. This phenomenon, called the self-mixing effect (SME), led to the discovery of a new class of laser interferometry which is called self-mixing interferometry (SMI or optical feedback interferometry (OFI) technology. SMI has the same resolution (half-wavelength resolution) as the conventional laser interferometry for displacement resolution. In 1978, this interferometry principle was experimentally demonstrated by Donati [2] using a gas laser to detect the Doppler shift caused by a moving remote target. The reported research work [3,4,5] has shown that SMI can achieve a high signal-to-noise-ratio and is very sensitive to the external cavity length detuning, with the aid of an internal photodetector attached to a laser diode. To date, SMI in lasers has been applied in various applications of sensing and instrumentation [3,4,5,6,7,8,9,10,11].

An SMI system needs to only consist of a laser diode (LD), a lens and an external target, hence it is simple and inexpensive in its implementation [2,3]. Generally, the LD is set at a constant bias current. When the external target moves, a conventional SM signal can be detected by a monitor photodetector (PD) packaged in the rear of the LD. The SM signals are characterized by optical feedback factor *C* whose definition can be found from [8,12], which is defined as follow:

(1) $$C=\frac{\kappa \tau }{\tau_{in}}\sqrt{1+\alpha^{2}}$$

where κ is the feedback coupling strength which is determined by the reflectivity of the laser emission facet and target facet, τ and $\tau_{in}$ are the laser roundtrip time in the external cavity and internal cavity respectively, α is the linewidth enhancement factor. At a weak feedback regime with C<1, a conventional SM signal contains sinusoidal-like fringe structure. At a moderate feedback regime, which is for C>1, a conventional SM signal shows asymmetric hysteresis and produces a sawtooth-like fringe structure. The mechanism of generating a conventional SM signal as well as its behavior with respect to C has been well established and presented in [3,4,12]. The mathematical model reported in the literature for describing such SM signal waveforms is derived from the steady-state solution of the Lang and Kobayashi (L-K) equations [13]. The model is valid under the assumption that both the electric field E(t) and carrier density N(t) in an LD with a stationary external cavity can reach a constant state after a transient period.

However, the work in [14,15,16] shows that the undamped relaxation oscillation (RO) or chaos may occur for an LD operating above its system stability boundary. In this case, E(t) and N(t) exhibit complicated oscillations rather than a constant level, we call the laser outputs RO-SM signals to differentiate them from conventional SM signals reported in literatures [2,3,4,6,7,8,9,10,11,12]. Obviously, the existing SMI model is not able to describe a RO-SM signal waveform. The behavior for an LD with optical feedback is generally described by a bifurcation diagram as a function of the feedback strength [17], from which a few characteristic states are identified for the laser intensity, including steady, periodic, quasiperiodic and chaotic oscillation. However, for sensing and measurement purposes (SMI systems), we wish to know how the laser intensity varies with the movement of an external target. We thus proposed to characterize the behavior of an LD in the plane of parameters C and the feedback phase (associated with the target movement), which are two important parameters for an SMI system [17]. Based on the different waveform features of the laser intensity, the work in [18] proposed three operational regions, with respect to C, to characterize an SMI system, referred to as stable, semi-stable and chaotic regions (we call it unstable below). The stable region is where the existing SMI systems operate. In the chaotic region, the movement information of a target cannot be found from the waveform of the laser intensity. However, when an LD operates in the semi-stable region (with the value of C above the stability boundary but below the chaotic level), the waveform of the laser intensity $E^{2}(t)$ exhibits a stable oscillation and may be capable of sensing the movement of the external target. The discovery presented in this paper is very important, as we found that, by both simulations and experiments, a SMI system can easily and sometimes inevitably enter into the semi-stable region, e.g., for the case when the feedback level C is around 2.5, which is quite commonly reported for the moderate feedback level in the literature. In this case, the usual detection approach by employing the PD packaged at the rear of an LD is not able to fully detect a RO-SM signal due to its limited bandwidth. Hence, when using a SMI system, it is necessary to know at which region the SMI system really operates. Note that the stability boundary for a practical SMI system can be determined by the method presented in [17].

In this paper, firstly, simulations are made for calculating the RO-SM signals based on the well-known Lang and Kobayashi (L-K) equations by choosing several sets of parameters corresponding to the LD status in the different regions named “stable”, “semi-stable” and “unstable”. Then the experiments are conducted on different LDs to verify the simulations. The features of RO-SM signals are studied in both time and frequency domains. The results show that RO-SM signals are highly sensitive to a moving target in contrast to conventional SM signals and thus have potential sensing applications.

## 2. Simulations and Experiments 

The dynamics of an LD with optical feedback can be described by the L-K equations [13], given below:

(2) $$\frac{dE(t)}{dt}=\frac{1}{2}\left\{G\left[N(t),E(t)\right]-\frac{1}{\tau_{p}}\right\}E(t)+\frac{\kappa }{\tau_{in}}\cdot E(t-\tau )\cdot \cos\left[\omega_{0}\tau +\phi (t)-\phi (t-\tau )\right]$$

(3) $$\frac{d\phi (t)}{dt}=\frac{1}{2}\alpha \left\{G\left[N(t),E(t)\right]-\frac{1}{\tau_{p}}\right\}-\frac{\kappa }{\tau_{in}}\cdot \frac{E(t-\tau )}{E(t)}\cdot \sin\left[\omega_{0}\tau +\phi (t)-\phi (t-\tau )\right]$$

(4) $$\frac{dN(t)}{dt}=J-\frac{N(t)}{\tau_{s}}-G\left[N(t),E(t)\right]E^{2}(t)$$

where $G\left[N(t),E(t)\right]=G_{N}\left[N(t)-N_{0}\right]\left[1-\varepsilon \Gamma E^{2}(t)\right]$ is the modal gain per unit time. The physical meanings for the symbols appearing in Equations (2)–(4) are listed in Table 1. The values of the symbols used in the simulations in this paper are also provided in the table. 

**Figure 1:**
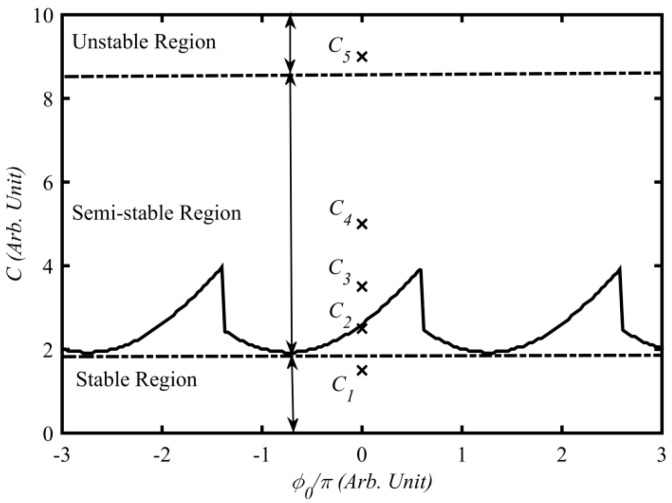
Improved stability boundary for describing an SMI system when $L_{0}=35\;\mathrm{cm}$, J=1.1Jth.

Based on above equations, the stability boundary can be obtained with respect to the external cavity parameters (*L*, *k*) and the injection current (*J*) using the method presented in [19]. Using the relationships presented in [18], we then can convert the stability boundary to the plane of ($\phi_{0}$, *C*), where $\phi_{0}$ is the light phase change in the external cavity which is related to the cavity length (*L*) and light speed (*c*) via $\phi_{0}=2\omega_{0}L/c$. The solid line in Figure 1 shows such a stability boundary when the injection current is 1.1 times the threshold current (J=1.1 Jth) and initial external cavity (*L*_0_) is 35 cm long. From Figure 1 we can see three regions, i.e., stable, semi-stable and unstable respectively.

**Figure 2:**
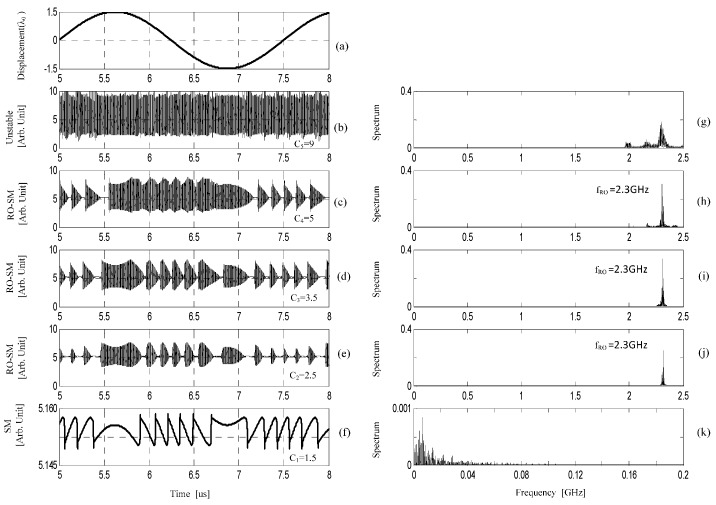
Modulated laser intensity at different regions and their corresponding spectra, where the laser intensity is scaled by $E^{2}(t)/10^{20}$ (**a**) displacement of the external target; (**b**–**f**) laser intensity when *C* = 9, *C* = 5, *C* = 3.5, *C* = 2.5, and *C* = 1.5 respectively; (**g**–**k**) spectra corresponding to (**b**–**f**) respectively. Note that the DC component in each case is removed when applying FFT.

In the following simulations, all the parameters are set according to Table 1. Under the same injection current (J=1.1 Jth) and initial external cavity length ($L_{0}=35\;\mathrm{cm}$), we vary the values of *C* so that the SMI system operates in the stable, semi-stable and unstable regions, e.g., *C* = 1.5, 2.5, 3.5, 5 and 9 corresponding to C1, C2, C3, C4 and C5 indicated in Figure 1. As we can see, C1 locates in the stable region, C2–C4 in the semi-stable and C5 in the unstable region. For all the cases, the external target moves in a simple harmonic form. Suppose that the initial cavity length is $L_{0}=35\;\mathrm{cm}$, and the displacement of the target is $\Delta L(t)=\Delta L_{m}\sin(2\pi f_{0}t)$, where, $f_{0}=400\;\mathrm{KHz}$, $\Delta L_{m}=1.5\;\lambda_{0}$, $\lambda_{0}=780\;\mathrm{nm}$. The corresponding waveforms of laser intensity $E^{2}(t)$ (called SM/RO-SM signals) are numerically solved from the above L-K equations and shown in Figure 2, where the laser intensity is scaled by $E^{2}(t)/10^{20}$. We then apply fast Fourier transform (FFT) on the signals to calculate their magnitude spectra as shown in the right column of Figure 2. Note that we removed the DC component from each signal before applying the FFT. Figure 2a shows the displacement of the target; Figure 2f shows the conventional SM signal located in the stable region which is commonly seen in the literature [2,4,7,8,10]; Figure 2c–e show the RO-SM signals in a semi-stable region; Figure 2b shows the signals in unstable region; and Figure 2g–k are spectra corresponding to the signals in Figure 2b–f respectively. From Figure 2, we can see the following:

1. The RO-SM signals exhibit the form of high frequency oscillation with its amplitude modulated by a slow-varying signal. Interestingly, the slow-varying envelopes are similar to the conventional SM signal characterized by the same fringe structure. It can be seen from the left column in Figure 2, that there are nearly six fringes corresponding to the peak-peak displacement ($3\lambda_{0}$) of the target. That is, each fringe in the RO-SM signals also corresponds to a target displacement $\lambda_{0}/2$, and hence the RO-SM can also be used to measure the displacement with the same resolution as the conventional SMI operating in the stable region.
2. Although having the same fringe structure, the RO-SM signals are very different from the conventional SM signals in their frequencies. The right column in Figure 2, shows that, the spectrum of a conventional SM signal (Figure 2k) locates in the relatively low frequency range, stopping at 0.2 GHz for the case with *C* = 1.5. However, the dominated frequency components associated with a RO-SM signal locate in a much higher frequency range, with a central frequency of 2.3 GHz (denoted by *f_RO_*). *f_RO_* is generated due to the relaxation oscillation when the SMI system operates above its stability boundary.
3. The Peak-Peak (P-P) value of a conventional SM signal (in Figure 2f) located in the stable region is around 0.012 while the P-P values of the RO-SM signals in semi-stable region are about 6.64, 5.04 and 4.12 respectively as shown in Figure 2c–e . Hence the RO-SM signals are much stronger (more than 300 times stronger) than the conventional SM signal. This implies that an SMI system working at a semi-stable region has potential for achieving sensing with improved sensitivity.
4. When the SMI system enters the unstable region shown in Figure 2b,g, the laser output will be unstable, characterized by a much wider frequency spectrum. In this case there is not an obvious relationship between the target movement and the laser output, and hence the system is not suitable for such waveform based sensing.

As described above, the amplitudes of the RO-SM signals carry the information of displacement, and hence detection of the amplitude can yield the latter. However, the RO frequency is very high, i.e., around 2.3 GHz based on our calculation, and hence we need to modify the conventional SMI system to capture such high-frequency signals. Generally, a conventional SM signal is observed by an internal monitor PD packaged at the rear of an LD. The time response of such PDs is very low and the bandwidth is less than a few hundred megahertz. Therefore, to detect the RO-SM signals, we have to employ a high-speed amplified photodetector. In the experiments described below, we have used a photodetector from Thorlabs (PDA8GS) with the bandwidth of 9.5 GHz as an external detector to pick up RO-SM signals.

**Figure 3:**
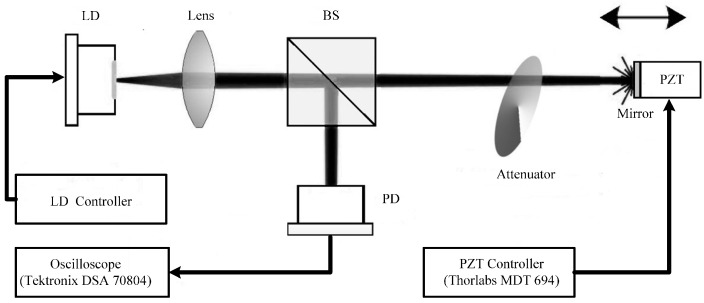
Experimental setup.

The experimental system is depicted in Figure 3. The laser source is a commercial LD (HL8325G, Hitachi, Tokyo, Japan), which is a single mode quantum well laser with an emitting wavelength of 830 nm and a maximum output power (*P*) of 40 mW. The LD is driven by a laser controller (LDC 2000, Thorlabs, Newton, NJ, USA) with the injection current being above the threshold (Jth=40 mA). The temperature of the LD is stabilized at 22 ± 0.01 °C by the temperature controller (TED 200, Thorlabs, Newton, NJ, USA). A mirror is used as the external cavity which is glued on the surface of a PZT actuator (PAS009, Thorlabs, Newton, NJ, USA) in order to provide sufficient optical feedback to the LD. The PZT actuator is driven by a PZT controller (MDT694, Thorlabs, Newton, NJ, USA). The displacement resolution of the PZT is 53 nm. A high-speed digital oscilloscope (DSA70804, Tektronix, Beaverton, OR, USA) with a sampling rate of 25 GS/s is used to observe RO-SM signals. The laser beam emitted from the LD is focused by a lens onto the mirror. A portion of the laser beam is then reflected back to the LD and causes SME. An attenuator is used to adjust the optical feedback level of the SMI system. A beam splitter (BS) with a splitting ratio of 50:50 is used to direct a part of the self-mixing light into the external fast PD through the fiber port coupler. The detected SM/RO-SM signals are then collected and recorded by the digital oscilloscope.

**Figure 4:**
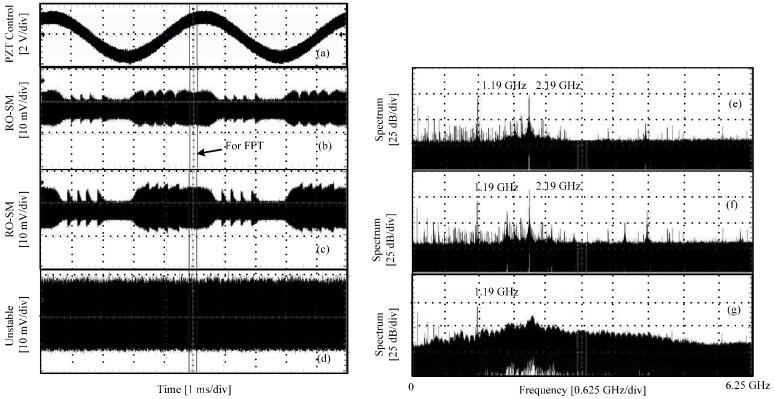
Experimental signals and their spectra in semi-stable and unstable regions. (**a**) PZT control signal; (**b**,**c**) RO-SM signals in semi-stable region; (**d**) SM signal in unstable region; (**e**–**g**) Spectra corresponding to (**b**–**d**) respectively.

It is difficult to conduct the experiments covering all three regions under the condition of the same injection current (*J*) and external cavity length (*L*). Hence, we designed two groups of experiments to confirm the phenomena predicted in the above simulations. In the first group, the injection current to the LD is 44 mA, and the initial external cavity length is 25 cm. In this case, we are able to observe the phenomena happened at semi-stable and unstable region. The relevant experimental steps are described as below:

1. Initially, set the PZT stationary, and adjust the attenuator so that an RO signal can be observed by the oscilloscope.
2. Apply a control signal to the PZT actuator using the PZT controller. The signal is a 30.0 V DC offset superposed with a sinusoidal voltage signal (200 Hz and 3.9 V P-P). The corresponding displacement (P-P) generated by the PZT is 2.08 μm.
3. Adjust the attenuator to vary the feedback strength from low to high, and record three RO/SM waveforms at different feedback levels, as shown in the left column in Figure 4. Meanwhile, for each case, the frequency spectrum is measured using the function provided by the oscilloscope. The corresponding frequency spectra are shown in the right column.

**Figure 5:**
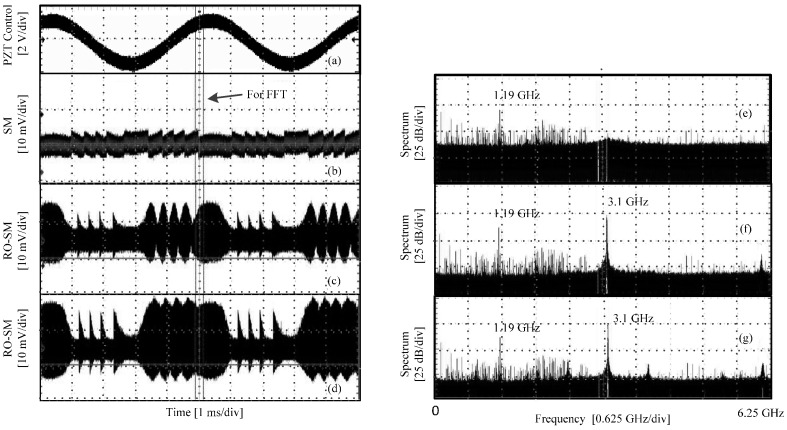
Experimental signals and their spectra in stable and semi-stable region. (**a**) PZT control signal; (**b**) conventional SM signals at stable region; (**c**,**d**) RO-SM signals at semi-stable region; (**e**–**g**) the spectra corresponding to (**b**–**d**) respectively.

Figure 4a shows the control signal for the PZT actuator; Figure 4b,c show the RO-SM signals when the LD is in a semi-stable region; Figure 4d shows the signal in the unstable region; and Figure 4e–g are the spectra corresponding to Figure 4b–d, respectively. From these waveforms we can see that the P-P value grows with the increase of the feedback strength. It can be seen that about five fringes can be found which correspond to the PZT displacement with 2.08 μm P-P. Hence, each fringe corresponds to 416 nm, which is about *λ*_0_/*2* of the LD. Note that, due to the use of a very high sampling frequency (i.e., 12.5 GS/s), we are only able to take a very short time duration (indicated in Figure 4b) of the signals for FFT. Hence, the spectrum in Figure 4 can only show where the central frequency (*f_RO_*) is. At the semi-stable region, a dominant frequency component can be found at the location of 2.19 GHz, as shown in Figure 4e,f. The envelope in the signal disappears in Figure 4d and its corresponding spectrum is significantly broadened. In this case, the SMI system is working in the unstable region. The observed phenomena from the experiments are consistent with the simulations results. Note that there exists a frequency component at 1.19 GHz across all the spectra shown Figure 4 and Figure 5; the reason for its existence will be explained below.

In the second group, we aim to compare the features of RO-SM and conventional SM signals in both time and frequency domains. By shortening the external cavity length and increasing the injection current, both stable and semi-stable regions can be achieved. In the experiments, we set the initial cavity to 14.5 cm and the injection current to 53 mA. The PZT is controlled in the same way as in group 1 of the experiments. The attenuator is adjusted to change the feedback level. Figure 5 shows the experimental signals recorded, from which we can see that the peak-peak value of a RO-SM signal is much higher than that of the SM signal in Figure 5b. The fringe number in a RO-SM is the same as the one in the conventional SM signal. Therefore, we confirm that the RO-SM signals have the same displacement resolution with conventional SM signals.

We also chose a short piece from each signal (marked in the left column in Figure 5) and measured the corresponding spectrum. It can be seen that a dominant frequency component located at $f_{RO}=3.1\;\mathrm{GHz}$ can be found in the spectrum of each RO-SM signal but not in the conventional SM signal. Again, the amplitude of RO-SM signals exhibits a similar fringe structure and hence carries the information of the target movement. As mentioned above, the frequency component at 1.19 GHz always exists in the spectrum, and it can be observed even when there is no input to the oscilloscope. Therefore, this 1.19 GHz component must be from the oscilloscope or other external disturbances rather than from the self-mixing effect.

**Figure 6:**
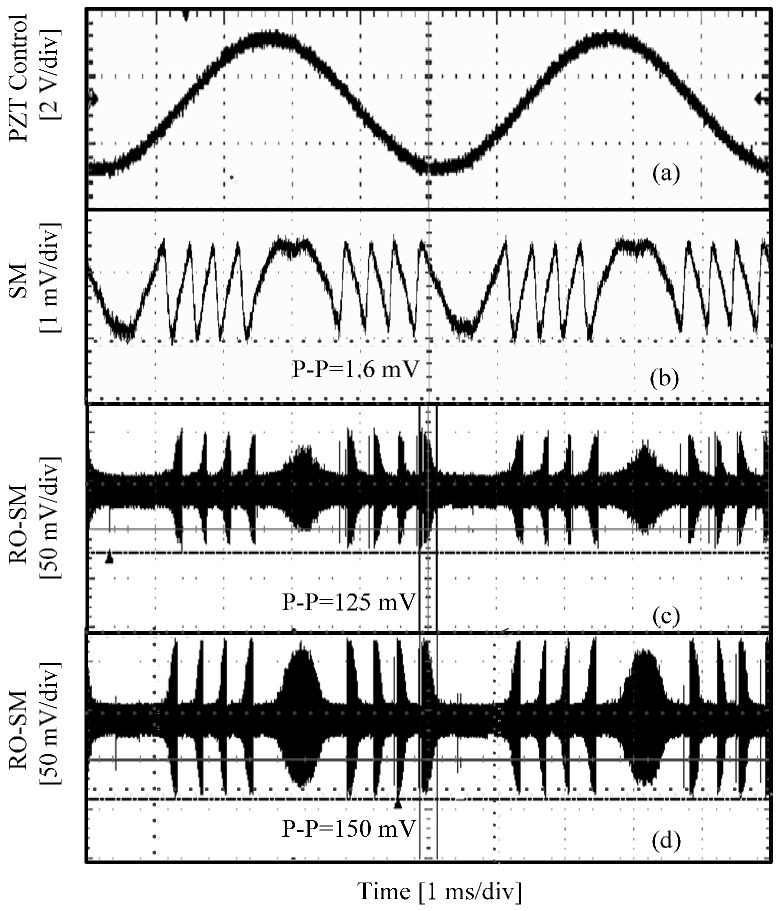
Experimental signals with *J* = 46 mA and *L*_0_ = 12.5 cm for DL5032-001 (**a**) PZT control signal; (**b**) conventional SM signals at stable region; (**c**,**d**) RO-SM signals at semi-stable region.

Another point we want to mention is that the signals in Figure 4 and Figure 5 are displayed by setting the oscilloscope at 10 mV/div, and hence the signals, including the 3.1 GHz and 1.19 GHz components, are all very weak indeed. This is because only a small amount light from the LD can be detected due to mismatch among the LD (HL8325G), the fiber coupler and the PD. In order to obtain stronger RO-SM signals, we also carried experiments using another two laser diodes. The first one was DL5032-001 (Jth=35 mA, *λ*_0_ = 830 nm, *P* = 30 mW) and we did the experiments under the conditions of *J* *=* 46 mA and *L*_0_ *=* 12.5 cm for both stable and semi-stable cases. The experimental results are presented in Figure 6. For the same target movement, we noticed that RO-SM signals are always much stronger (about 93 times higher) than a conventional SM signal.

**Figure 7:**
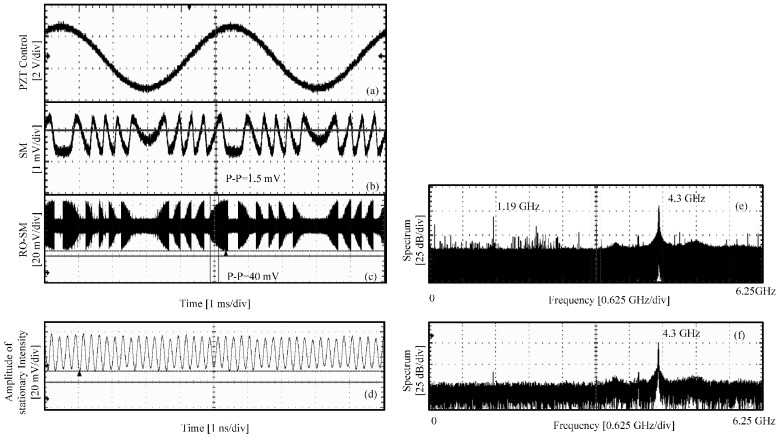
Experimental signals for DL4140-001S. (**a**) PZT control signal; (**b**) conventional SM signals at stable region; (**c**) RO-SM signals at semi-stable region; (**d**) the laser intensity when the target is stationary; (**e**,**f**) the spectra corresponding to (**c**,**d**) respectively.

Another laser diode we tested was DL4140-001s (*J*th = 30 mA, *λ*_0_ = 785 nm, *P* = 20 mW). The results are shown in Figure 7. The conventional SM signal is obtained with *L*_0_ = 11 cm, *J* = 60 mA and the RO-SM signal with *L*_0_ = 14 cm, *J* = 46 mA. It shows that the P-P value of RO-SM signal is 27 times that of the conventional SM signal. The spectrum obtained for a small piece from the RO-SM is shown in Figure 7e with *f_RO_* = 4.3 GHz. In the semi-stable region, we also recorded the RO signal and its spectrum is shown in Figure 7d,f, respectively, when the target is stationary. It can be seen that *f_RO_* is same as the one in Figure 7e. This implies that an RO-SM signal has a central frequency that is the same as the relaxation oscillation of the LD system with optical feedback.

In summary of the above, we can say that the experiments are consistent with the numerical simulation by solving the L-K equations.

## 3. Conclusions

This paper reveals the new phenomena for an LD with external optical feedback where the LD operates at a semi-stable region which is in between relaxation oscillation and chaos. Such a phenomenon can be observed by using a photodetector with a high-frequency bandwidth. To the best of our knowledge, it is the first time that these phenomena are reported and the details of a RO-SM waveform are described. A RO-SM signal exhibits high-frequency oscillation with its amplitude modulated by a slow time-varying envelop. This slow time-varying envelop is characterized by a similar fringe structure as the conventional SM signals, and hence it can be used for displacement measurement with the same fringe resolution. Furthermore, in contrast to a conventional SM signal, a RO-SM signal is found to be much stronger, 300 times stronger in simulations and 93 times stronger in experiments on DL5032-001. Hence, we say an RO-SM signal has potential for achieving sensing with improved sensitivity.

## Figures and Tables

**Table 1:** Meanings of the symbols in the L-K equations.

Symbol	Physical Meaning	Value
t	time	
E(t)	amplitude of the intra-cavity electric field	
ϕ(t)	phase of the intra-cavity electric field, $\phi (t)=\left[\omega (t)-\omega_{0}\right]t$	
ω(t)	laser angular frequency with feedback	
$\omega_{0}$	Laser angular frequency without feedback	$2.42\times 10^{15}{\;\mathrm{rads}}^{-1}$
N(t)	carrier density	
$G_{N}$	modal gain coefficient	$8.1\times 10^{-13}{\;m}^{3}\cdot s^{-1}$
$N_{0}$	carrier density at transparency	$1.1\times 10^{24}{\;m}^{-3}$
ε	nonlinear gain compression coefficient	$2.5\times 10^{-23}{\;m}^{3}$
Γ	confinement factor	3
$\tau_{p}$	photon life time	$2.0\times 10^{-12}\;s$
κ	feedback strength	
$\tau_{in}$	internal cavity round-trip time	$8.0\times 10^{-12}\;s$
α	line-width enhancement factor	6
J	injection current	
$\tau_{s}$	carrier life time	$2.0\times 10^{-9}\;s$
τ	light roundtrip time in the external cavity, τ=2L/c	
L	external cavity length	
